# Inhibition of ERR*α* Aggravates Sepsis-Induced Acute Lung Injury in Rats via Provoking Inflammation and Oxidative Stress

**DOI:** 10.1155/2020/2048632

**Published:** 2020-07-02

**Authors:** Wenfang Xia, Zhou Pan, Huanming Zhang, Qingshan Zhou, Yu Liu

**Affiliations:** ^1^Department of Critical Care Medicine, Renmin Hospital of Wuhan University, Wuhan 430060, China; ^2^Department of Cardiology, Renmin Hospital of Wuhan University, Wuhan 430060, China; ^3^Cardiovascular Research Institute, Wuhan University, Wuhan 430060, China; ^4^Hubei Key Laboratory of Cardiology, Wuhan 430060, China

## Abstract

Inflammation and oxidative stress are critical pathologies that contribute to sepsis-induced acute lung injury (ALI). This study investigated the regulatory role of estrogen-related receptor alpha (ERR*α*) in an experimental model of sepsis-induced ALI. *In vivo*, a cecal ligation and puncture- (CLP-) induced ALI model was established in anesthetized rats. Animals were then randomly assigned to receive an intraperitoneal injection of vehicle or ERR*α* inverse agonist (XCT-790, 2.5 mg/kg). Administration of XCT-790 significantly aggravated a sepsis-induced increase in pathological damage of lung tissues, lung endothelial permeability, myeloperoxidase (MPO) activity in lung tissues, production of serum inflammatory factors, and inflammatory cell accumulation in bronchoalveolar lavage fluid. In addition, XCT-790 treatment exacerbated a CLP-induced decrease in lung superoxide dismutase and an increase in lung malondialdehyde levels. *In vitro*, the exposure of rat pulmonary microvascular endothelial cells (PMVECs) to lipopolysaccharide (LPS) resulted in increased endothelial permeability and reduced expression of tight junction protein ZO-1, Occludin, JAM-A, and adherens junction protein VE-cadherin, which were further deteriorated by knockdown of ERR*α*. In addition, LPS-triggered inflammatory factor production and increase in the expression of phosphorylated I*κ*B*α* and NF-*κ*B p65 were also exacerbated by silencing ERR*α* gene. Meanwhile, knockdown of ERR*α* dramatically promoted LPS-activated mitochondrial reactive oxygen species production and LPS-induced downregulation of Sirt3 protein levels in rat PMVECs. Taken together, our present study provides evidences that ERR*α* functions as a novel negative modulator of sepsis-induced ALI in rats. The underlying mechanisms responsible for ERR*α*-elicited effects are largely dependent on the regulation of inflammatory response and oxidative stress.

## 1. Introduction

Sepsis is a dysregulated systemic response to infection that causes organ dysfunction. The lung is the most susceptible target organ in the case of sepsis, and acute lung injury (ALI)/acute respiratory distress syndrome (ARDS) can occur in the early stage of sepsis [1]. Despite the implementation of a lung-protective ventilation strategy, ALI/ARDS remains an important clinical problem associated with significant morbidity and mortality [2]. Therefore, there has been intense interest in elucidating the underlying molecular mechanisms and discovering novel therapeutic targets for sepsis-induced ALI.

Pulmonary microvascular leakage is one of the distinguishing characteristics of barrier dysfunction in septic ALI/ARDS [3]. The pathophysiology of endothelial barrier dysfunction is characterized by complex mechanisms that involve inflammatory response and oxidative stress [4, 5]. Lipopolysaccharide (LPS) of gram-negative bacteria has been suggested to act as endotoxin and bind its receptor on the cell membrane, initiating a series of innate immune responses. Exposure to LPS can induce rapid and robust production of inflammatory factors and reactive oxygen species (ROS) and subsequently disrupt the endothelial barrier, contributing to microvascular leakage [6, 7]. Therefore, signaling molecules modulating the inflammatory response and oxidative stress are crucial for preventing and treating sepsis-induced ALI.

Estrogen-related receptors (ERRs), the first discovered orphan nuclear receptors, are highly homologous to estrogen receptors in sequence. Estrogen-related receptor *α* (ERR*α*) was the first identified orphan nuclear receptor, with a crucial role in regulating energy metabolism and mitochondrial biogenesis [8, 9]. Yuk et al. found that the expression of ERR*α* was strongly upregulated by stimulation with various toll-like receptor agonists in macrophages [10]. Furthermore, they demonstrated that ERR*α* acts as a negative regulator of toll-like receptor-induced inflammatory response through enhanced activation of NF-kappaB (NF-*κ*B) signaling [10]. In addition, previous studies have shown that ERR*α* is required for the induction of mitochondrial reactive oxygen species (ROS) production and mice lacking ERR*α* are susceptible to Listeria monocytogenes infection [11]. Another study found that suppressing the activity of ERR*α* by its inverse agonist XCT-790 in 3T3-L1 adipocytes led to a significant increase in ROS production [12]. A recent study revealed that ERR*α* deficiency exacerbated cisplatin-induced renal dysfunction and tubular injury, as well as oxidative stress [13]. Although these reports provide insight into the potential role of the ERR*α* in inflammatory response and oxidative stress, the regulatory role of ERR*α* in an experimental model of sepsis-induced ALI is unclear. In the study described here, we aimed to investigate the role and underlying mechanisms of ERR*α* in the regulation of sepsis-induced ALI.

## 2. Materials and Methods

### 2.1. Animals and Sepsis Model

Forty-eight male Sprague-Dawley rats (220-280 g) were purchased from the Hubei Provincial Laboratory Animal Public Service Center (Wuhan, China). All experiments were approved by the Animal Care and Use Committee of Renmin Hospital at Wuhan University and confirmed the Guide for the Care and Use of Laboratory Animals published by the US National Institutes of Health (the 8th Edition, NRC 2011).

Rats were randomly divided into the Control (Ctr) group, Sham group, Sepsis group, and Sepsis+XCT-790 intervention group. Sepsis was induced by cecal ligation and puncture (CLP) as previously described [14]. Briefly, rats were anesthetized with 2% pentobarbital sodium (25 mL/kg) by intraperitoneal injection. Then, the cecum was ligated and punctured twice with a 20-gauge needle. Sham control animals underwent the same procedure without CLP. Animals in the Sepsis+XCT-790 group received 2.5 mg/kg XCT-790 (Sigma, USA) 30 minutes before CLP intraperitoneally, and animals in the Sepsis group received the same volume of normal saline. All animals were subcutaneously resuscitated with normal saline (3 mL/100 g body weight) immediately after surgery. At 24 hours after CLP, the experiment was terminated.

### 2.2. Pathological Examination

Lung tissues were fixed with 4% paraformaldehyde for hematoxylin and eosin staining. Lung morphologic changes were observed by light microscopy. The degree of pathological injury was scored based on edema, neutrophil infiltration, hemorrhage, and disorganization of lung parenchyma, as a previously scoring system described [15]. The degree was graded numerically from 0 to 4. Higher scores indicate more severe lung damage.

### 2.3. Lung Vascular Permeability Assay

Lung vascular permeability was evaluated by measuring Evans blue dye leakage. Briefly, 1% Evans blue dye (2 mL/kg) was injected intravenously into rats and allowed to circulate for 3 minutes. Then, the pulmonary artery was cannulated and perfused with saline. The left atrium was opened with an incision to allow for drainage effluent. The lungs (100 mg) were then removed and homogenized. The homogenate was incubated in formamide (1 mL/100 mg) for 24 hours at 37°C and then centrifuged for 10 minutes at 4000 g. The supernatants were collected, and the absorbance was determined by spectrophotometric analysis at a wavelength of 630 nm.

### 2.4. Assessment of Lung Myeloperoxidase (MPO) Activity

For estimates of the numbers of neutrophils in tissue, the left lung lobe was homogenized and processed as previously described for the determination of MPO activity [16]. MPO activity was expressed as units MPO/mg protein in the tissue homogenate using the Bio-Rad DC protein assay (Bio-Rad, Hercules, CA) for protein determinations following detergent solubilization of MPO.

### 2.5. Measurement of Serum Inflammatory Factor Levels and Inflammatory Cell Influx into the Airways

Rats were anesthetized, and blood sample was collected. The blood was centrifuged at 2000 rpm and 4°C for 10 minutes, and the supernatants were separated for tumor necrosis factor-*α* (TNF-*α*) and interleukin-1*β* (IL-1*β*) determination using enzyme immunoassay kits according to the operation manual of the kits. For inflammatory cell count in bronchoalveolar lavage fluid (BALF), the right main bronchus was clamped, and then, the left lung was lavaged three times by instillation of 4 mL PBS solution. BALF was immediately harvested and centrifuged at 2000 rpm and 4°C for 10 min to collect the cell pellet. Inflammatory cell count in BALF was performed using a standard hemocytometer.

### 2.6. Detection of Lung Superoxide Dismutase (SOD) Activity and Malondialdehyde (MDA) Levels

The SOD activity was detected by the WST-1 Cell Proliferation Assay kit, while the MDA levels were determined using a total bile acid colorimetric assay in the lung homogenates according to the manufacturer's instructions (Jiancheng Biotech Ltd., Nanjing, China). The activity of SOD was quantized by absorbance at 550 nm and was expressed in units of U/mg protein. The concentration of MDA was measured by absorbance at 523 nm and was expressed in units of nmol/mg protein.

### 2.7. Cultured Pulmonary Microvascular Endothelial Cells and *In Vitro* Endothelial Permeability Assays

Rat pulmonary microvascular endothelial cells (PMVECs) were pursued from Bei Na Chuanglian Biotechnology Research Institute (Beijing, China). PMVECs were cultured in Dulbecco's modified Eagle's medium (DMEM), with 10% fetal bovine serum (Gibco, Invitrogen, Carlsbad, USA) at 37°C in a humidified atmosphere of 5% CO_2_. When the cells reached 80%-90% confluency, they were passaged with trypsin-ethylenediaminetetraacetic acid (EDTA; Gibco). The cells were then inoculated into a 6-well plate (3 × 10^5^ per well) when the cultured cells were fused to about 50%. To knockdown ERR*α* expression, we infected PMVECs with recombinant lentiviral shERR*α* (RiboBio Co., Ltd, Guangzhou, China) at a multiplicity of infection of 10. Endothelial permeability was determined by the horseradish peroxidase (HRP) permeability assay, as previously described [17]. After 2 h, 6 h, and 12 h, HRP activity was measured with a microplate spectrophotometer at OD450.

### 2.8. Measurement of Inflammatory Factor Levels

The PMVECs were stimulated with LPS (10 *μ*g/mL), then the culture supernatants were collected at 6 and 12 hours, centrifuged at 4°C for 15 min, and the supernatant was stored at -20°C. The levels of TNF-*α* and IL-1*β* in the cell culture supernatant were measured by enzyme-linked immunosorbent assay (Beyotime, China) and performed according to the kit instructions.

### 2.9. Evaluation of Mitochondrial ROS Levels

Mitochondrial ROS levels were determined using MitoSOX-Red (Molecular Probes), as previously described [18]. Dilute the 5 mM MitoSOX reagent stock solution in PBS to make a 5 *μ*M MitoSOX reagent working solution. Then, apply 1.0 mL of 5 *μ*M MitoSOX reagent working solution to cover cells adhering to coverslips. Incubate cells for 10 minutes at 37°C, protected from light. Wash cells gently three times with warm buffer. Samples were viewed under a fluorescence microscope.

### 2.10. Western Blotting

Lung tissues were collected at 24 hours after CLP for Western blotting analysis. The PMVECs were stimulated with LPS for 12 h and then washed with PBS (4°C) and lysed using RIPA buffer. Western blot analysis of ERR*α* (Cell Signaling Technology), NF-*κ*B p65 (Abcam), IkB*α* (Santa Cruz), p-NF-*κ*B p65 (Cell Signaling Technology), p-IkB*α* (Cell Signaling Technology), ZO-1 (Abcam), Occludin (Cell Signaling Technology), JAM-A (Abcam), VE-cadherin (Cell Signaling Technology), Sirt3 (Santa Cruz), *β*-Actin (Servicebio), and GAPDH (Abcam) was performed according to standard protocols. All immunoblots were scanned and quantified using the Odyssey Infrared Imaging System.

### 2.11. Statistical Analysis

Data are expressed as mean ± SD. Statistical analysis was performed using SPSS 11.0 statistic software. Differences among the different groups were analyzed for statistical significance by one-way ANOVA, followed by the LSD-*t* test. A *P* value of <0.05 was considered statistically significant.

## 3. Results

### 3.1. Inhibition of ERR*α* Aggravated CLP-Induced Lung Injury and Lung Vascular Hyperpermeability

**Figure 1:**
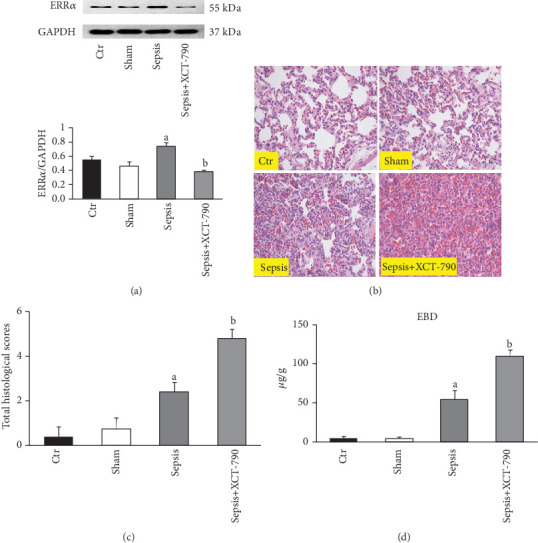
Inhibition of ERR*α* aggravated CLP-induced lung injury and lung vascular hyperpermeability. (a) ERR*α* expression levels in the four groups. Representative blots (upper panel); Quantitative results (lower panel). (b) The lung tissues were histologically analyzed by hematoxylin and eosin in different groups (×200). (c) Lung injury was assessed by histological scores for each group. (d) Comparison of lung vascular Evans blue leakage in different groups. The values are expressed as mean ± SD. ^a^*P* < 0.05 compared with the Ctr and Sham group; ^b^*P* < 0.05 compared with the Sepsis group.

To investigate the potential role of ERR*α* in sepsis-induced lung injury, we first examined whether ERR*α* expression levels were altered in lung tissue in septic rats. Western blotting showed that ERR*α* expression was dramatically upregulated in lung tissue of septic rats compared with controls, which was suppressed by the treatment of XCT-790 (Figure 1(a)). As shown in Figure 1(b), no pathological changes were seen in the Control group and the Sham group; however, there were serious injuries in the Sepsis group. After administration of XCT-790, lung injury was exacerbated to different degrees.  Figure 1(c) showed that the histological scores were significantly higher in the Sepsis group than that in the Control group and the Sham group (*P* < 0.01). Administration of XCT-790 aggravated the lung injury as suggested by increasing histological scores. In addition, sepsis led to significant lung vascular hyperpermeability as indicated by a prominent increase in vascular Evans blue leakage, which was also enhanced after treatment with XCT-790 (Figure 1(d)).

### 3.2. Inhibition of ERR*α* Elevated Lung MPO Activity, Serum TNF-*α* and IL-1*β* Levels, and Inflammatory Cell Count in BALF

**Figure 2:**
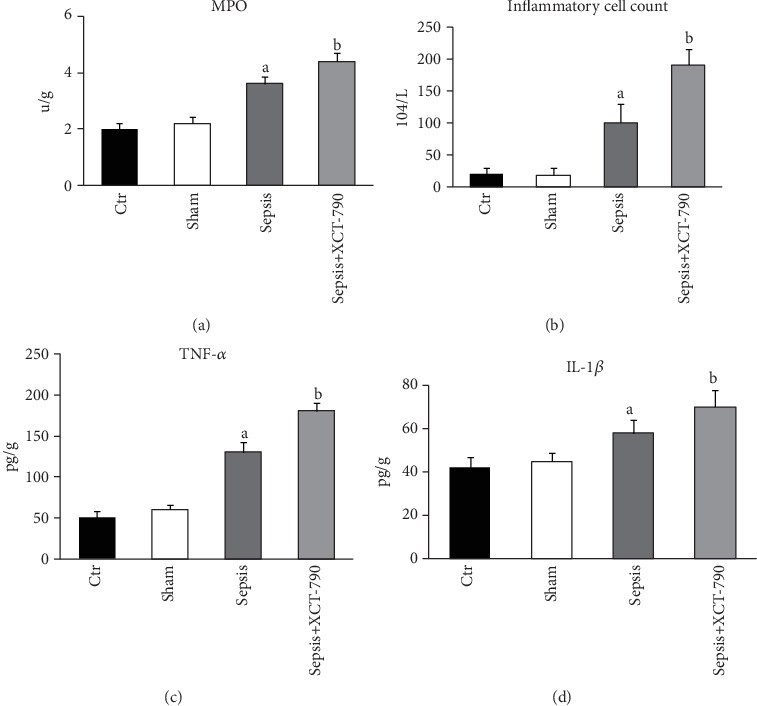
Inhibition of ERR*α* elevated lung MPO activity, serum TNF-*α* and IL-1*β* levels, and inflammatory cell count in BALF. (a) Comparison of lung MPO activity in different groups. (b) Comparison of inflammatory cell count in BALF in different groups. (c, d) Comparison of serum TNF-*α* and IL-1*β* levels in different groups. The values are expressed as mean ± SD. ^a^*P* < 0.05 compared with the Ctr and Sham group; ^b^*P* < 0.05 compared with the Sepsis group.

Compared with the Control group and the Sham group, lung MPO activity was significantly increased. However, the administration of XCT-790 further elevated lung MPO activity (Figure 2(a)). Similarly, there was excessive inflammatory cell infiltration into the lungs in the Sepsis group compared with that in the Control group or the Sham group, which were further increased in rats treated with XCT-790 (Figure 2(b)). In the Control group and the Sham group, serum TNF-*α* and IL-1*β* were maintained at a low level. After CLP, serum TNF-*α* and IL-1*β* levels were dramatically increased, suggesting the massive release of inflammatory factors. More importantly, the production of TNF-*α* and IL-1*β* was notably evaluated when XCT-790 was administered (Figures 2(c) and 2(d)).

### 3.3. Inhibition of ERR*α* Decreased Lung SOD Activity and Increased MDA Production

**Figure 3:**
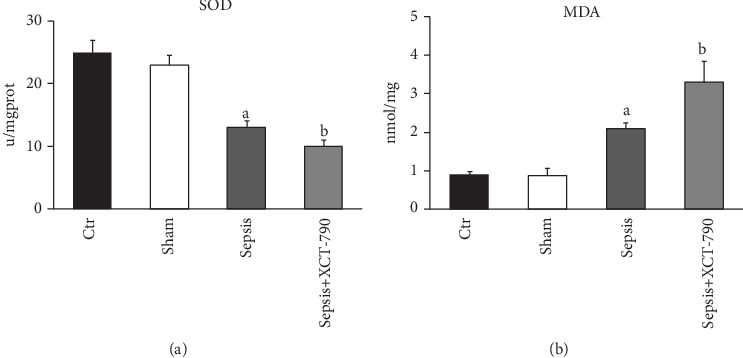
Inhibition of ERR*α* decreased lung SOD activity and increased MDA production. (a) Comparison of lung SOD activity in different groups. (b) Comparison of lung MDA content in different groups. The values are expressed as mean ± SD. ^a^*P* < 0.05 compared with the Ctr and Sham group; ^b^*P* < 0.05 compared with the Sepsis group.

The results shown in Figure 3 reveal that CLP caused a significant increase in MDA content and reduction in SOD activity in lung tissues compared with those in the Control group and the Sham group (*P* < 0.05). However, treatment with XCT-790 markedly increased CLP-induced MDA production and decreased SOD activity in rats with CLP-induced ALI (*P* < 0.05).

### 3.4. Knockdown of ERR*α* Deteriorated LPS-Induced Endothelial Hyperpermeability in PMVECs

**Figure 4:**
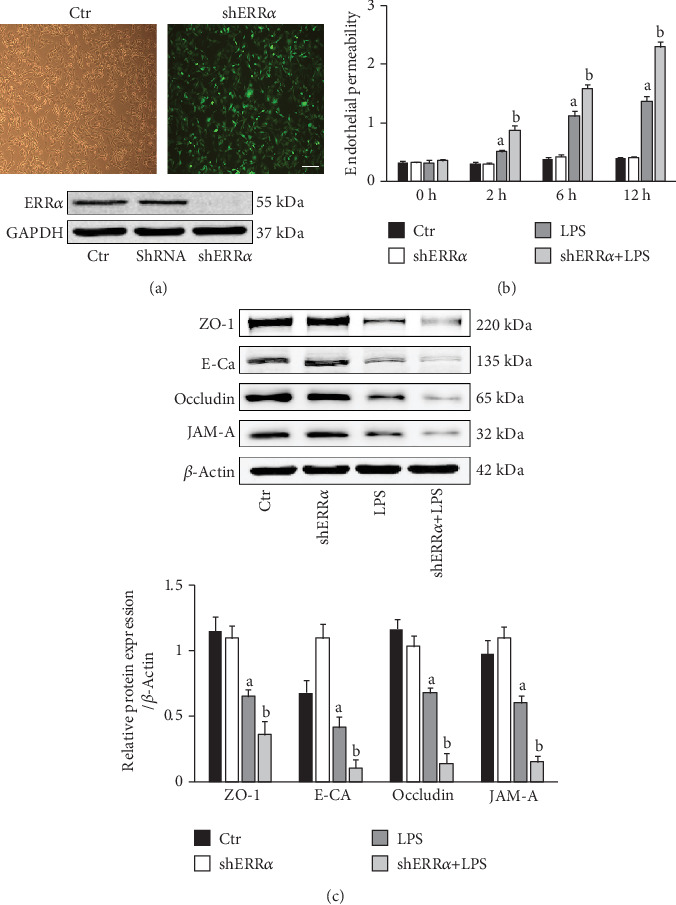
Knockdown of ERR*α* deteriorated LPS-induced endothelial hyperpermeability in PMVECs. (a) Representative images were taken to observe the cell morphology of normal rat PMVECs (upper left panel) and the expression level of green fluorescent protein GFP (upper right panel) observed under an inverted fluorescence microscope after 72 hours of infection. Bar = 50 *μ*m. The expression levels of ERR*α* were detected by Western blot (lower panel). (b) Comparison of endothelial permeability in the four groups at different time points. (c) Representative blots of ZO-1, Occludin, JAM-A, and VE-cadherin in the four groups (upper panel); quantitative results of ZO-1, Occludin, JAM-A, and VE-cadherin (lower panel). The values are expressed as mean ± SD. ^a^*P* < 0.05 compared with the Ctr and shERR*α* group; ^b^*P* < 0.05 compared with the LPS group.

As shown in Figure 4(a), the morphology of rat PMVECs was fusiform or polygonal (upper left panel). PMVECs cells were infected with MOI = 10, and the expression of green fluorescent protein was observed under a fluorescence microscope (upper right panel). Western Blot results showed that the expression levels of ERR*α* in the shERR*α* group were significantly reduced (lower panel). Compared with the control cells, treatment of the PMVECs with LPS *in vitro* resulted in a significant increase in endothelial permeability. However, ERR*α* gene silencing remarkably deteriorated LPS-induced PMVEC hyperpermeability (Figure 4(b)). Western blot analyses showed that LPS stimulation led to a notable decrease in the expression of tight junction proteins ZO-1, Occludin, JAM-A, and adherens junction protein VE-cadherin, which were exacerbated by knockdown of ERR*α* (Figure 4(c)).

### 3.5. Knockdown of ERR*α* Accelerated LPS-Induced Inflammatory Factor Release in PMVECs

**Figure 5:**
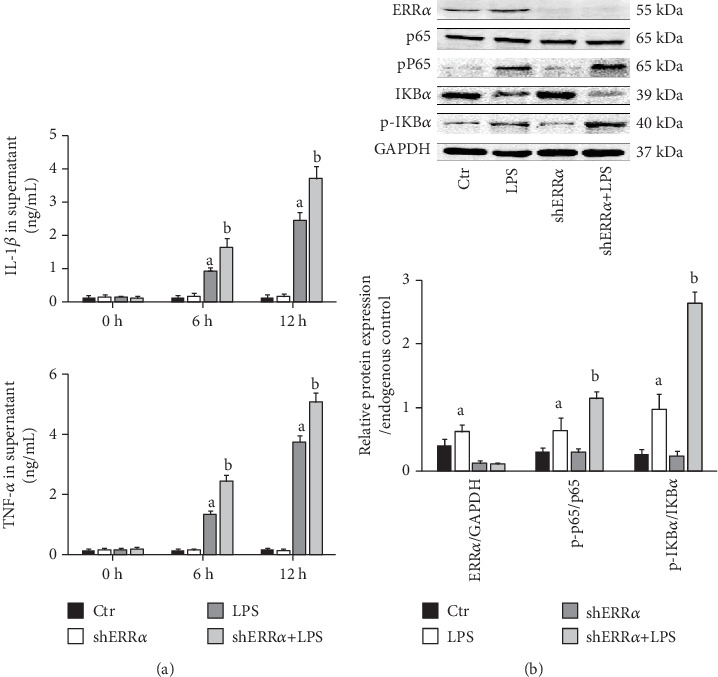
Knockdown of ERR*α* accelerated LPS-induced inflammatory factor release in PMVECs. (a) Comparison of TNF-*α* and IL-1*β* levels in different groups at different time points. (b) Representative blots of ERR*α*, p65, p-p65, IKB*α*, and p-IKB*α* in the four groups (upper panel); quantitative results of ERR*α*, p65, p-p65, IKB*α*, and p-IKB*α* (lower panel). The values are expressed as mean ± SD. ^a^*P* < 0.05 compared with the Ctr and shERR*α* group; ^b^*P* < 0.05 compared with the LPS group.

Compared with the Control group, the concentration of TNF-*α* and IL-1*β* in the LPS group was significantly increased at 12 h after LPS stimulation. However, knockdown of ERR*α* further elevated the TNF-*α* and IL-1*β* levels (Figure 5(a)). To explore the possible mechanism underlying the regulatory role of ERR*α* in LPS-induced inflammatory response, we next examined the NF-*κ*B signaling pathway. As expected, the expression of p-p65 and p-IKB*α* in the LPS group was significantly higher than that in the Control group after LPS stimulation. Importantly, knockdown of ERR*α* significantly increased the expression of p-p65 and p-IKB*α* compared with the LPS group (Figure 5(b)).

### 3.6. Knockdown of ERR*α* Enhanced LPS-Induced ROS Production in PMVECs

**Figure 6:**
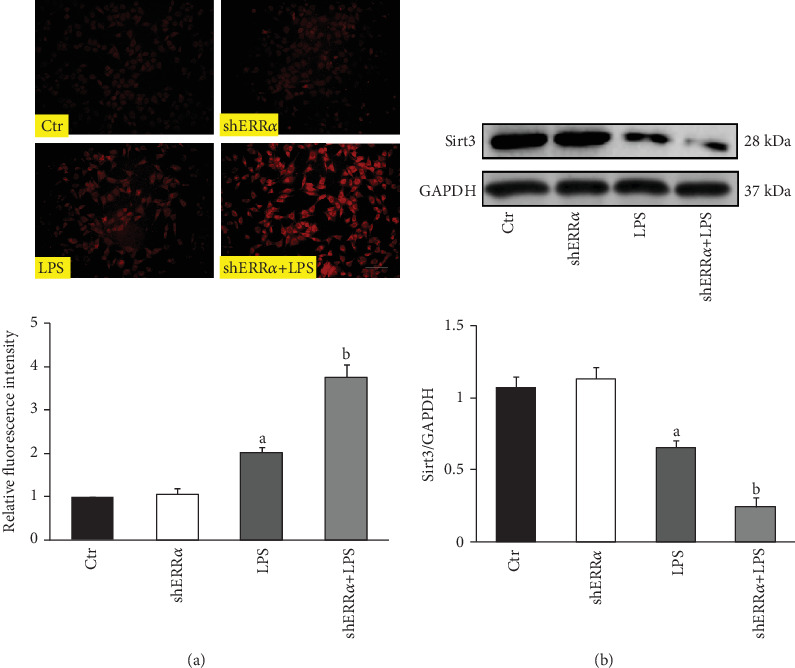
Knockdown of ERR*α* enhanced LPS-induced mitochondrial ROS production in PMVECs. (a) Detection of mitochondrial ROS levels in PMVECs using a MitoSOX Red superoxide indicator. The red fluorescence indicated the presence of superoxide in the treated groups, whereas the control cells showed minimal fluorescence (upper panel). Bar = 50 *μ*m. The relative fluorescence intensity is compared among different groups (lower panel). (b) Representative blots of Sirt3 in the four groups (upper panel); quantitative results of Sirt3 (lower panel). The values are expressed as mean ± SD. ^a^*P* < 0.05 compared with the Ctr and shERR*α* group; ^b^*P* < 0.05 compared with the LPS group.

Compared with the Control group, the mitochondrial ROS concentration was significantly higher in the LPS group after LPS stimulation, which was further increased in the ERR*α* knockdown group (Figure 6(a)). Western blot analyses showed that LPS stimulation significantly reduced the protein levels of Sirt3, and knockdown of ERR*α* further decreased the expression of Sirt3 (Figure 6(b)).

## 4. Discussion

In the present study, we reported for the first time that ERR*α* negatively regulated the development of sepsis-induced ALI both *in vivo* and *in vitro*. Pharmacological inhibition of ERR*α* or knockdown of ERR*α* significantly aggravated sepsis-induced lung pathological damage and vascular endothelial hyperpermeability. Mechanistically, ERR*α*-elicited effects on sepsis-induced ALI are largely dependent on the regulation of inflammatory response and oxidative stress.

It is considered that the essence of ALI is an excessive and uncontrolled inflammatory response. Extensive lung inflammation contributes to the destruction of the basement membrane and increased the permeability of the alveolar-capillary membrane [19]. In this study, we found that CLP induced significant neutrophil infiltration in lung tissue and increase in lung MPO activity and inflammatory cell count in BALF. The release of proinflammatory mediators, such as TNF-*α*, IL-1*β*, and IL-6, has been demonstrated to play a critical role in the early phase of ALI. Arbibe et al. found that endotoxin-induced increase in the expression of type II phospholipase A2 in macrophages during acute lung injury was prevented by the administration of anti-TNF-*α* antibody [20]. Wolthuis et al. confirmed that etanercept, a recombinant human soluble TNF receptor fusion protein, reduced the number of neutrophils in BALF and attenuated ventilator-induced lung injury [21]. In the present study, the production of TNF-*α* and IL-1*β* was markedly increased by CLP or LPS challenge, which was further aggravated by pharmacological inhibition of ERR*α* or knockdown of ERR*α*. Our results suggested the anti-inflammatory effects of ERR*α* both *in vivo* and *in vitro*. NF-*κ*B signaling plays a central role in tissue inflammatory and immune response via transcriptionally regulating gene expressions [22]. Previous studies have demonstrated that NF-*κ*B is one critical transcription factor required for maximal expression of many cytokines involved in the pathogenesis of acute lung injury [23]. A previous study found that the absence of ERR*α* augmented diethylnitrosamine-induced activation of the NF-*κ*B pathway in Kupffer cells, resulting in an increase in the inflammatory response [8]. In addition, ERR*α* deficiency promoted NF-*κ*B signaling and led to excessive systemic and macrophage inflammatory responses [10]. These results suggest that there is molecular crosstalk between ERR*α* and NF-*κ*B signaling. In the present study, we observed that LPS challenge induced the degradation of I*κ*B-*α* and activation of NF-*κ*B *in vitro*, and the activation of NF-*κ*B signaling was further elevated by ERR*α* knockdown, suggesting that activation of NF-*κ*B signaling plays an important role in the deteriorative effects of ERR*α* knockdown on ALI.

Increasing evidence shows that excessive oxidative stress is thought to play an essential role in the pathogenesis of ALI. Excessive ROS generation impairs endothelial function and promotes lung inflammation, and similarly, inflammatory cells can lead to an overproduction of ROS, which creates a vicious cycle of worsening ALI [24]. Previous studies have demonstrated that ERR*α* is an important regulator of ROS production [11–13]. In this study, we observed that XCT-790 markedly increased CLP-induced MDA production and decreased SOD activity in rats with CLP-induced ALI. Consistently, LPS elicited significant oxidative stress and triggered ROS generation *in vitro*, which was further enhanced by ERR*α* knockdown. PGC-1*α* is a transcriptional coactivator that acts as a central regulator of energy metabolism. It has been found that PGC-1*α* is a broad and powerful regulator of ROS metabolism [25]. It has been shown that ERR*α* interacts with transcriptional coactivator PGC-1*α* that bound to the Sirt3 promoter as its transcription factor to regulate Sirt3 expression [26]. Previous studies suggested that Sirt3 is the primary mitochondrial deacetylase, limiting the generation of mitochondrial-derived ROS [27]. In this study, the protein expression of Sirt3 was significantly reduced in response to LPS stimulation, which was further downregulated by knockdown of ERR*α*, indicating that the knockdown of ERR*α*-exhibited deteriorative effect on LPS-induced ALI may be closely correlated to inactivation of the Sirt3 signaling pathway.

There are some limitations in the present study. First, we only examined the role of ERR*α* on sepsis-induced ALI in male rats. However, whether there is a sex difference in the response to the inhibition of ERR*α* is unknown. Second, we used the drug XCT790 as an investigational tool, as it functions as a specific inhibitor of the ERR*α*. However, XCT-790 has been shown to be a potent mitochondrial electron transport chain uncoupler independent of its inhibition of ERR*α* [28]. Therefore, XCT-790 may exert a partial deleterious effect independent of its inhibition of ERR*α*. Finally, the present study suggests that inhibition of ERR*α* exerts a deleterious effect on sepsis-induced ALI. However, we did not evaluate the effect of ERR*α* activation on sepsis-induced ALI.

In summary, it may be concluded that ERR*α* functions as a novel negative modulator of sepsis-induced ALI in rats. Mechanistically, ERR*α*-elicited protective effects are mediated through the inhibition of inflammatory response and oxidative stress.

## Data Availability

The data used to support the findings of this study are available from the corresponding author upon request.
